# The Treatment of the Irregularities of the Teeth

**Published:** 1893-01

**Authors:** Eugene S. Talbot

**Affiliations:** Chicago, Ill.


ARTICLE II.

### THE TREATMENT OF THE IRREGULARITIES OF THE TEETH.

BY EUGENE S. TALBOT, M. D., D. D. S., CHICAGO, ILL.

Read before the annual meeting of the Dental Society of the State of New York, May 11, 1892.

In the preparation of a paper upon this subject for a meeting like this, it is difficult to decide what to say and what not to say, because it covers so much ground. What I shall say however, will be upon phases that I hope will draw out discussion.

According to a well-known authority,* one of the earliest writers to mention treatment of irregularities of the teeth was Egenolff, in 1541. To show the vast stride of advance made since then, I will quote from the translation of his pamphlet: "It often happens that to children more than seven years of age, when the teeth begin to drop out, other teeth grow by the side of those which are about to fall out; therefore we should loosen the tooth about falling out from the gums, and move it often to and fro until it can be taken out, and then push the new one every day toward the place where the first one was, until it sets there and fits among the others; for if you neglect to attend to this the old teeth will remain, become black, and the young one will be impeded from growing straight and can no more be pushed to its right place."

It is evident from these remarks that it is only within the last three hundred years that much thought has been given to this subject, and that the slight knowledge of treatment for irregularities at that time and for some time subsequently was of a prophylactic nature; that is, it was confined entirely to the management of the teeth of the first set, with the view of proper eruption of the second set. Evidently only such irregularities as were being caused by the deciduous teeth were regarded as within the power of man to correct. Could it be that there were not as many such cases then as now? I have examined large collections of old skulls in Europe and America, and found no V or saddle arches, or any variations of them. It would seem that this is an indication that perhaps these forms of irregularities are of comparatively modern origin.

In previous papers I have shown that irregularities of the teeth are now on the increase. I have also shown that there seems to be no plan by which this increase can be checked, unless it be the care of children's teeth, and the observance of common sense rules regarding extraction of the adult teeth.

---

*Farrar's Treatise on Irregularities.

While dentistry has been practiced in this country for more than a century, I have been unable to find a single mention of the correction of any of the constitutional forms of irregularities prior to the year 1859, at which time mention was made by Dr. Westcott in an article upon the subject of what he denominated "Expansion of the Upper Dental Arch." * Brief mention had been made of the cause of these deformities, however, as early as 1825.† This shows that while the very able practitioners at that period were well versed in theories and the plans of operations then in vogue, they had written little and accomplished less in the line of invention of mechanisms for the correction of such deformities. In this alone, may we not see some evidence that aberrations in the jaws and teeth were then uncommon, or else that they were regarded as impossible to correct? Since that time, however, we know that knowledge of treatment for correction of irregularities has increased rapidly, and that it has now become a specialty.

The subject of irregularities of the teeth and their correction is a branch of surgery that requires special attention, special education, special training. In 1878, Dr. Farrar, feeling that the importance of this branch of dentistry was not appreciated, made considerable effort to show that it should be taught to greater extent in our professional schools. Indeed, he tried to urge upon some of the members of the faculties of two or three colleges the matter of establishing a special chair upon the branch, but at the time no one except himself appeared to feel that there was sufficient to the subject of irregularities of the teeth and their correction to warrant any such effort. Since that time a great change has taken place in professional opinion; so great is this change that I believe that if the time has not already come, it is not far off when every first class dental college will have such a chair.

---

* Dental Cosmos, September, 1859; American Journal.

† See "Irregularities of the Teeth," (Talbot).

I have frequently observed cases that were disfigured and ruined for life, by incompetency. Probably many persons present have seen similar cases. Three cases that were mismanaged have come under my care during the past twelve months; the first one a woman of fine appearance, who had lost her upper lateral incisors and called upon a dentist for treatment. He attempted to draw the cuspids into close proximity to the central incisore. The woman had given her entire vacation and all her money for this operation, and then it was only partially successful. That such an operation was wrong needs but little argument. The expression of the mouth was pointed, and the features were otherwise distorted. Had a small plate bearing lateral incisors been inserted in the space, instead of having had the cuspids moved, the facial expression would have been improved instead of injured.

Case No. 2 is that of a young man with what seemed at first and what an old practitioner had regarded as a "prognathous lower maxilla." This I found upon inspection to be caused by arrested development of the upper maxilla. This dentist had spent five years in trying to reduce the deformity. It was fortunate, however, that he was unsuccessful, for had he succeeded the deformity would have been made greater than it was. Instead of moving the lower teeth back, the upper teeth should have been moved forward. In six months' treatment by this plan, the teeth were correct and the face greatly improved.

The third case was one that occurred in my early practice. To make room for the anterior teeth to be carried backward, I extracted a tooth upon one side only. The result was that the incisors moved around to that side, causing an unsightly appearance. Fortunately, however, I have been able to correct this condition by extracting one on the other side. These mistakes are frequently made by dentists. Of course, I do not wish it inferred that two teeth should be taken in all cases. As one author* suggests, "the relation of the line between the centrals to the median line of the face should determine in the main the question as to whether one or two teeth should be extracted, because sometimes the centrals are at a considerable distance to one side of their proper place. The nose is generally the best guide for determining this esthetic question, the exceptions being when the nose is at one side of the median line of the face. Even then the septum of the nose should influence this matter, as well as the median line of the face."

From these cases, as samples of many, we see that to correct irregularities of the teeth we should have a broad knowledge of the human body. It is not sufficient that a dentist understands mechanics and physics; he should have considerable knowledge of high art. In truth, the specialist should be what may be called a scientific man,—well read in evolution, anthropology, ethnology, as well as in anatomy of the human face. No dentist ever lived whose intellect was too great or whose education was too extensive for the profession of dentistry. Of course he should understand physiology, pathology, and especially the subject of absorption and deposition of new matter, and the influence that the condition of the patient has upon these two processes, and also the unfavorable results that may arise from too mnch irritation from moving the teeth too rapidly. He should also have the kind of education that will enable him to observe any derangement of the system that may result from strain,—a result that is especially liable to occur in delicate children at an age when teeth can be moved most easily. Such knowledge will better enable him to discover the cause of the deformity, and in many cases aid him in deciding the kind of treatment that will best correct that deformity. Not long ago I heard of a young lady being confined to her house, and part of the time to her bed, for two years, as a

---

* J. N. Farrar.

result of having her teeth regulated. This case was treated by a dentist who thinks it is not necessary to know anything about general medicine in order to practice dentistry, and he is a teacher, too.

Irregularities of the teeth cannot always be prevented. There are, however, two causes of irregularity that might be prevented, that which results from lack of proper care of the deciduous teeth, and that from extraction of the first adult molars. I regard these two sources as great causes of arrest of development of the jaws. I mean the alveolar ridges and the dental arches. We are all aware that the deciduous teeth generally decay early, and that they are frequently extracted long before the proper time. Some dentists do not feel the importance of preserving the deciduous teeth until they are to be soon replaced by their successors. The object of preserving deciduous teeth is to prevent the second teeth from erupting upon their territory. Especially is this true of the first adult molars, which, if not prevented from moving forward, will encroach upon the space necessary for the second bicuspids.

It has been the practice with some dentists to teach pupils that it is proper to extract these first molars, because "the jaw is crowded;" that "by the extraction of these four teeth, room will be made for the remainder to appear in line." One dentist said that his father invariably removed these teeth for the purpose of preventing irregularities, and that it would be impossible to find a single person over seven years of age in his neighborhood that could show a first "permanent" molar. I think that the habit of extracting the first adult molars during the past forty years has done more to cause arrest of development of the alveolar ridge than any other thing.

In reply to a letter written to a dentist in Glasgow, requesting him to "measure the distance from one first molar across the arch to the opposite molar," he said that if he tried he would not be able to find three hundred adults in his whole country who would have their first molars. Another dentist of many years' experience has lately published in this country an article advocating the extraction of these first molars as a means of prevention of irregularities of the teeth. Thus it will be seen that even in this late day the followers of this pernicious habit still exist.

In a few cases such extraction might be beneficial, but generally it must lead to disastrous results. I regard these first molars as fixed bases in the dental arch, and that the position of the other teeth depends much upon them for forcing the teeth and alveolar process outward and forward to the proper line. The effect of the constant habit of extracting these adult molars in a family is arrest of development of the alveolar process, which in time, I think, will cause inherited smallness of the jaws.

The extraction of these teeth sometimes causes another kind of irregularity, which, though not unsightly when the jaw is seen independent of its mate, is sometimes, so far as the utility of the teeth is concerned, more disastrous. I allude not only to the inclining forward of the second molar and an inclining backward of the bicuspids, but of the contraction of the dental arch, so that it not only impairs the antagonism, but destroys it almost entirely. I have seen cases, as probably most of you have, in which the extraction of the upper first molars has caused the remaining teeth to form an arch as small as to occlude inside of the lower arch, leaving no teeth to antagonize except the corners of the extreme posterior molars.*

The space required to correct an irregularity is never so large as that made by the extraction of the two first molars, and seldom is it necessary to have more space than can be obtained by the extraction of one bicuspid on each side. As these latter teeth are generally situated

* Some of the bad results of extracting these teeth are well shown in a recent paper by Dr. Davenport in the "Dental Cosmos."

nearer the point of the greatest degree of irregularity, the operation is often facilitated by their extraction. Of course I do not advocate extraction of bicuspids, or any other teeth in all cases of overcrowding, but when it is necessary to extract side teeth at all, I prefer as suggested by one authority, to extract a sound bicuspid rather than a first adult molar, even though the latter should be considerably decayed.

In a paper upon the subject of "The Differentiation of Anterior Protrusions of the Upper Maxilla and Teeth," read before the last International Medical Congress, I explained how the different varieties of protruding teeth were caused, and remarked that if these lesions are thoroughly understood by the operator, it will enable him to correct them easier than if he does not understand them. For illustration, protrusions caused by arrest of development, or those from socket disease, are easier to reduce than those protrusions caused by deposition of bone cells, such for instance, as are illustrated in Dr. Kingsley's work, page 131.* In the first mentioned varieties we have simply normal alveolar process to resorb by the application of pressure upon the teeth. But in a patient twelve years of age, with the lower incisors antagonizing with the gums in the anterior part of the valut of the mouth, the result of long teeth or of excessive height of the alveolar ridge, the upper incisors protrude between the lips at an angle of 45 degrees. We have an alveolar process as dense and hard as true bone, and the pressure required to move the teeth must be several times greater, often greater than can be obtained from any anchorage that can be made in the mouth.

APPARATUS.—Special plans of treatment have been advanced by dentists, and special kinds of apparatus have been invented and put forward in the market for general

* "Oral Deformities" These cases are also referred to in my work, page 161.

use, but it is rare to find one of these that will serve for more than one case, even though it be of the same class, without considerable alteration or without being all made over. Many practitioners of dentistry have been misled by advertisements of these goods. They read well, but they are a delusion and a snare. I think that those devices which are recommended for reducing anterior deformities caused by arrest of development of the maxillæ should be used with caution, or else the incisors and cuspids, instead of being moved backward, will stand still, and the anchor teeth (the bicuspids and molars) move forward. One author,† however, in his work shows to what extent the side teeth are useful to anchorage, and also points out when to resort to the head-gear. In my hands the only satisfactory appliance in all cases is the skull-cap of Fox, making the back of the head anchorage.

The degree of difficulty in such cases depends somewhat upon the shape and thickness of the alveolar process, height of vault, and the age of the patient.

It may be claimed that the devices on the market are only approximate in form; that the operator is expected to make alterations before putting them into use. But often it would require more skill to alter such ready made mechanism' than it would to invent and construct new cases. No matter how well he may understand the cause of a deformity and the device which is necessary to correct the case, unless he knows how to construct apparatus so that it will meet the needs of each case, he will be liable to fail as a regulator. Frequently dentists send me models and ask for opinions as to the best plan of correcting the cases. They often also call for the mechanism for correcting them. My plan of reply is to return the models and refer the senders to certain well known forms of apparatus, naming them in the order which they should be considered. If one will not meet the demands of the

---

† J. N. Farrar.

case, another can be studied. I purposely refrain from speaking upon any special plans of treatment, unless I can have the opportunity to personally study the mouth and face.

During the past ten years special devices have been urged, through private and public clines, upon the profession by different operators, as special "systems" of practice. As a proof of the value of their "systems," they show cases in plaster which at first glance appear to show favorable results, but to an expert many of them show unfavorable results. The profession has but to examine the illustrations in some of our monthly journals to find such failures. Some of these things have even been patented, not for protection of the profession, but for making money out of dentists. If these things, some of which are old to the profession, were practicable when bought, there would be some redeeming feature in the business. I have tried all that are on the market, with one exception, and I have not yet found one case for which I could not devise something different that would accomplish the work more easily and with better results. The exception referred to is a device that would be worthless for any case. Show me a dentist who tries to regulate all cases with one piece of apparatus, or one set of them, and I will show you an unsuccessful practitioner. Do not misunderstand me. Most mechanisms made for sale may have accomplished the desired results in the cases for which they were devised, but the assertion that they will meet the needs of all similar cases I think is unsound. I believe that every one who has had experience with them will say that "they have been tried and found wanting." Many operators, including myself, who have been deluded by these advertisements, have sometimes not only been disappointed in the thing itself, but have been drawn into embarrassing positions with patients.

I have already alluded to such a case in the treatment of anterior protrusions, when the posterior teeth have been carried forward instead of the anterior teeth being carried backward.

The treatment for correction of irregularities should be like that of true medicine. It should not restrict itself to any one thing or any one plan, but selected from all things and from all plans that have proven efficient. It may, of course, require sound judgment to select that which is the best for a case, but that does not prove anything unsound in my remark.

Every operator in this branch of dentistry should have large resources at his command from which to select the best materials for regulating mechanisms for any given case, because there is no panacea.

To Dr. Norman W. Kingsley, in 1880, is due the credit of introducing several effective devices, and credit for bringing together in a section of his valuable work (principally devoted to other subjects) much of that which was then known upon the subject of irregularities. Since the issue of this book another work on the science and art of the treatment of the irregularities of the teeth has been published by a different author. This reflects great honor upon the dental profession, not only by the vastness of the work, but because the author has handled the various phases of the different subjects in a highly scientific way and in a catholic spirit. The volume is so well systematized that any dentist referring to it can find information on almost every conceivable point, and also find illustrated almost every valuable mechanism that has ever been used for the correction of any kind of a case or class of cases. At the risk of being regarded as enthusiastic, I will say that this encyclopedia—for that is what it really is—will always stand out in bold relief as one of the few grandest blossoms of the dental profession, and as a product developed in our generation we should be proud of it. To say in Dr. Kingsley's words that Dr. Farrar's book "is the most elaborate and exhaustive work of its kind," is not too great an encomium.

At the present day I think the dentists should discard clumsy plates whenever it is possible, and I think it probable that in the near future, as has been demonstrated in the past fifteen years by some of the ablest specialists in this branch, nearly all irregularities, including the most difficult forms, will be treated with skeleton mechanisms consisting of clamp-bands, ferrules, levers, gold ribbons, gold and steel screws, and rubber and wire springs. Plates, however, have their uses, and are sometimes absolutely necessary to success, but when plates are called for they should be as light and small as possible consistent with effectiveness.

From the time that the first treatments for correction of teeth were reported by different dentists, up to about the year 1876, all or nearly all mechanisms consisted of plates and narrow strips of sheet gold, or silver, placed along the dental arch, and the teeth were connected with them by strings or rubber rings. The plates as then made were cumbersome, filthy, and the strings tied on the teeth cut deeply into the gums, causing irritation, inflammation, and pain.

To Dr. W. H. Dwinelle, of New York City, is due the credit of making in 1849 one of the greatest advancements in the treatment of widening the dental arch, the use of the screw-jack. This was made of steel. It reminded for Dr. Farrar, however, to substitute steel screw-jacks by gold screw jacks, and to make and develop numerous plans for using screws in other ways as aids in causing continued as well as intermittent force, with the least harm possible to the tissues. In 1866 the introduction of steel piano wire in combination with plates, invented by P. Headridge, of England, and two years later developed by the elder Coffin and his son, was another stride in advance over earlier plans of using springs; but in Dr. Farrar's combination of springs with screw-clamp bands for anchors, we have devices that are superior.

Wire, from its electricity and quality, enabling it to be bent in any desirable form, is available for use in many conditions of irregularities not before thought possible by anything. The use of clamp bands does away with clumsy plate anchors. For the best interests of patients of nearly or quite full growth, I have become convinced that when it is practicable to apply intermittent acting apparatus, it is by far the best. But there are many cases for which screws are not practicable, and wire springs and elastic rubber are better, and, strange as it may seem to some of you, for more than a hundred of this class of devices we are indebted to Dr. Farrar's ingenuity.

A very simple little device which I use constantly, and by which I reduce many abnormalities, is made of German silver rolled (Nos. 24-26 and 28, U. S. gauge) and cut into strips, and then bent in and out between the teeth; the ends being shortened every day. This device can be used between any of the teeth including the bicuspids and molars.

Considerable was said years ago about "jumping the the bite," as it is called, and a number of articles have appeared this past winter in the dental journals in which this subject has been revived. As I understand this phrase, it consists in throwing the lower jaw forward one-half the width of a bicuspid, thus bringing the lower incisor closer to the posterior sufaces of the upper incisors, and also causing the chin to become more prominent. In a case like this one or two conditions exist: either the upper jaw has developed beyond its normal position, or the lower jaw is arrested in its development. The latter is most likely the case in a large majority of cases. In either case the articulation at the joint is in a normal condition. I have never been able to "jump the bite," although I have tried it in a number of cases. I do not believe anyone else has been able to accomplish it, nor do I believe such a thing is possible. Were such a thing possible, one of two things must take place. First, absorption and deposition and deposition of bone cells must take place at the weakest part of the jaw, namely, at the angle, to allow of the forward movement, which is out of the question, for two reasons: First, there is not pressure enough; and second, if it could take place, the articulation of the teeth would be spoiled. Second, there must be a forward movement by absorption of the condyle in the glenoid cavity of the same amount of space to correspond to the forward movement of the teeth, which is out of the question for three reasons: First, there is not pressure enough to produce the absorption; second, tissue in front of the cavity would become involved, and third, nature would not construct a new cavity to accommodate the condyle.

FEES.—No one thing has a tendency to degrade this branch more than the plan of making fees for the operation. It is the custom with some dentists to examine a case and then make an estimate upon the operation, just as a carpenter estimates the cost of making a door or a window. In the latter case the carpenter knows what he is figuring upon, and therefore is sure to make a profit by his contract. On the other hand, the dentist may not be able to forejudge in regard to the extent of work necessary to complete an operation (because he has no means by which he may calculate upon the idiosyncrasies of his patients), therefore a set fee is an uncertain remuneration. If he values his time on the same basis as for other operations, the results are often too small. Frequently the operator is satisfied with small compensation, arguing that his time would not be otherwise used. This argument, of course, applies only to those who have a limited practice. A large proportion of the dentists practice their calling as if it were a trade, rather than as a profession. In no other department of surgery would the operator be satisfied with the fee for the time spent, as does the average dentist on regulating cases.

The method of a prominent Boston dentist is as follows: After taking the impression, a time is set for the next visit. This gives the operator an opportunity to study the case, as well as to inform himself as to the responsibility of the patient. When the patient returns a contract is agreed upon, and the basis is, say, one, two or three dollars per visit, and one, two, five, or ten dollars for each device used, the price being governed partly by the nature of the appliance to be made. The operator should never commit himself as to the nature of the devices to be used or to the length of time required for completion of the operation, for it may be necessary to change the device or plans of procedure two or three times before he is able to accomplish the desired results, or the operation may take more time than he at first supposed. By this plan patients and relatives are not disappointed, nor do they lose confidence in the ability of the operator.

In this way people of limited means are not deprived of the best services and the rich are charged no more than the poor. By this plan the dentist is better satisfied for services rendered than he usually is.—*Dental Cosmos.*

